# Metabolomic and Proteomic Changes in *Candida albicans* Biofilm in Response to Zosteric Acid Treatment

**DOI:** 10.3390/ijms232214067

**Published:** 2022-11-15

**Authors:** Cristina Cattò, Laura Corte, Luca Roscini, Gianluigi Cardinali, Federica Villa, Francesca Cappitelli

**Affiliations:** 1 Department of Food Environmental and Nutritional Sciences, Università degli Studi di Milano, 20133 Milano, Italy; 2 Department of Pharmaceutical Sciences-Microbiology, Università di Perugia, 06121 Perugia, Italy

**Keywords:** zosteric acid, antibiofilm, natural compounds, mechanism of action, metabolomics, proteomics, *Candida albicans*

## Abstract

Zosteric acid (ZA) is a secondary metabolite of the seagrass *Zostera marina,* with antibiofilm activity against fungi. Information concerning its mechanisms of action is lacking and this limits the development of more potent derivatives based on the same target and activity structure. The aim of this work was to investigate the ZA mode of action by analyzing the metabolic status of *Candida albicans* biofilm and its protein expression profile upon ZA treatment. Fourier-Transform Infrared Spectroscopy confirmed that ZA modified the metabolomic response of treated cells, showing changes in the spectral regions, mainly related to the protein compartment. Nano Liquid Chromatography–High-Resolution Mass Spectrometry highlighted that 10 proteins were differentially expressed in the *C. albicans* proteome upon ZA treatment. Proteins involved in the biogenesis, structure and integrity of cell walls as well as adhesion and stable attachment of hyphae were found downregulated, whereas some proteins involved in the stress response were found overexpressed. Additionally, ZA was involved in the modulation of non-DNA-based epigenetic regulatory mechanisms triggered by reactive oxygen species. These results partially clarified the ZA mechanism of action against fungi and provided insight into the major *C. albicans* pathways responsible for biofilm formation.

## 1. Introduction

Fungi form recalcitrant biofilms in many settings, with relevant consequences in terms of health, social and economic impact [1]. Within the biofilms, fungi are organized in complex communities associated with the surfaces and embedded in a self-produced polymeric matrix. The biofilm lifestyle offers protection against a wide range of environmental stresses as well as enhanced survival during harsh conditions. Indeed, in the form of biofilms, microorganisms are more tolerant to antimicrobial agents by up to several orders of magnitude, making their removal from surfaces difficult or even impossible [2]. In addition, several biocides have been banned as hazardous to the environment and human health [3]. Nowadays, the emphasis is placed on sustainable approaches that exploit the power of natural products to deprive microorganisms of their ability to develop biofilms, in a non-toxic way and with modalities that decrease the selection pressure for drug-resistant mutations [4].

**Figure 1:**
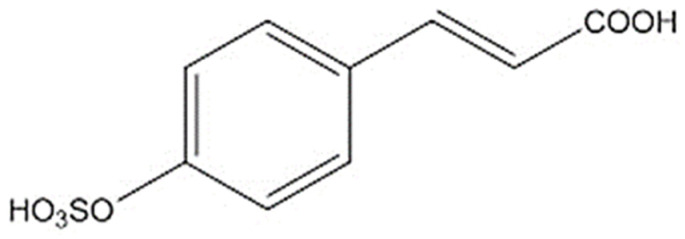
Chemical structure of ZA.

Zosteric acid (ZA) or p-(sulphooxy)cinnamic acid (Figure 1) is a secondary metabolite of the seagrass *Zostera marina* and it has been shown to be a powerful non-biocidal antibiofilm compound effective against bacteria, fungi and marine macrofouling [5,6,7]. Concerning fungal biofilms, Stanley and colleagues [8] reported that ZA reduced the adhesion of *Colletotricum lindemuthianum* and *Magnaporthe grisea* by 40% on both abiotic and plant leaves’ surfaces. Similarly, Villa and coauthors [9] demonstrated that ZA was effective against *Aspergillus niger* and *Penicillium citrinum*, with a reduction in spore adhesion of up to 57%. In addition, *Candida albicans* adhesion and the subsequent biofilm formation was reduced by at least 70% when treated with 10 mg/L ZA, on both hydrophilic and hydrophobic surfaces [5]. Cryosectioning of different biofilms combined with microscopy observations also revealed a significant impact of ZA on fungal biofilm thickness and morphology, i.e., leading cells to the inability to form filamentous structures, while remaining metabolically active. In addition, ZA-treated *C. albicans* biofilms displayed enhanced sensitivity to various antimicrobial agents, such as cis-2-decenoic acid, hydrogen peroxide, chlorine and chlorhexidine [5].

ZA application in real settings is very promising due to its low toxicity towards organisms and the environment. ZA showed a half-life of a few days in seawater, no measurable LD50 for larval fish and an acute toxicity profile similar to sugar [10]. Ecotoxicological tests did not find direct toxicity effects toward *Daphnia magna* [11]. Moreover, ZA showed a high solubility in water and a low octanol–water partitioning coefficient, indicating a low bioaccumulation capability in the aquatic compartment [11]. Cellular activity, adhesion, proliferation and morphology of both the murine fibroblast line L929 and the human osteosarcoma line MG-63 were not compromised after 10 mg/L ZA exposure [5].

Another important feature of ZA is its simple chemical structure, which allows one to produce this natural drug by chemical synthesis, at controlled concentrations and with an adequate level of purification. Indeed, a sustainable way to obtain this compound, avoiding the harvesting of its primary source (plants), has already been developed [9].

The challenge now is to take this knowledge and develop novel ZA-based tools for managing detrimental fungal biofilms. A bottleneck limiting the full exploitation of ZA for treating fungal biofilms is the general lack of information concerning its mechanism of action and cellular receptors. The understanding of the nature of ZA-target binding is a crucial step to (i) implement any ZA-based applications and materials, (ii) generate more potent derivatives based on the same structure–activity relationship and (iii) identify functional markers that can be used for the fast screening of other bio-based drugs against fungi [12]. Although the ZA mechanisms of action against bacterial biofilms are partially understood [6,13,14,15], the ZA mode of action against fungi remains unknown.

In this study, a high-level approach is proposed in order to greatly advance the current knowledge about the ZA mechanisms of action against fungal biofilms.

*C. albicans* bears unique features regarding its genome structure, cell biology, life cycle and adaptation to environmental challenges [16]. Moreover, several studies have also shown how biofilm-forming ability is a key feature to grant an environmental advantage to *C. albicans* in colonizing a lot of different settings, from the food industry to hospitals [17]. These features make *C. albicans* an attractive unicellular model to study genome biology and dynamics in eukaryotes [16].

In this research, *C. albicans* was chosen as the fungal model to investigate the ZA mode of action, by using a lab-scale model system able to simulate conditions encountered in vivo. Fourier-Transform Infrared Spectroscopy (FTIR) was successfully used as a metabolomic fingerprinting tool to describe the metabolic status of *C. albicans* cells in the presence of ZA. Additionally, proteins differently expressed in *C. albicans* biofilms upon ZA treatment were investigated by using the nano Liquid Chromatography–High-Resolution Mass Spectrometry (nLC-HRMS) suitable to scan the entire *C. albicans* proteome and provided, for the first time, the protein targets of ZA in fungal cells.

## 2. Results

### 2.1. Metabolomic Analysis Revealed a Leading Proteic Contribution in the Reaction to ZA

**Figure 2:**
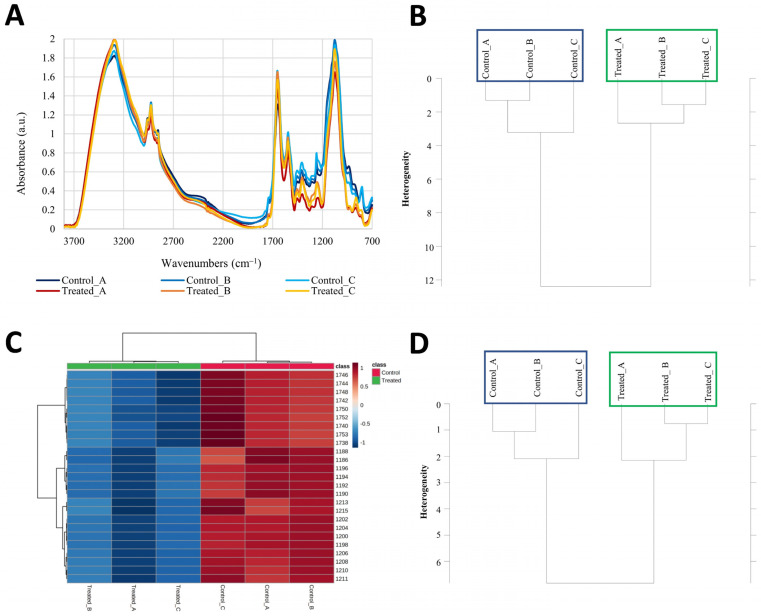
Representative IR spectra, Hierarchical Cluster Analysis (HCA) and heatmap of the top significantly altered region in IR spectra of control and ZA-treated biofilms. (**Panel A**): Representative average IR spectra of control (blue-shaded) and treated (red-shaded) biofilm samples; (**Panel B**): HCA carried out on whole raw spectra. (**Panel C**): Heatmap highlighting the most altered FTIR regions; (**Panel D**): HCA carried out on combined W_2_ and W_3_ regions.

Hierarchical cluster analysis of whole raw FTIR spectra of biofilm (Figure 2A,B) showed that ZA exposition induced a clear difference between the metabolomic response of control and treated samples, reaching a maximum heterogeneity value of about 12 a.u., six-times higher than the heterogeneity found within replicates (ca. 2 a.u.).

To define the cellular components responsible for this differentiation, a correlation analysis was carried out on the MetaboAnalyst 5.0 platform, using the VIP scores from PLS-DA (Figure 2C). Differentially altered wavenumbers belonged to W_2_ and W_3_ spectral regions, i.e., those containing mainly the IR peaks associated with the protein compartment of the cells and allowed for a neat clusterization into two groups of the analyzed samples. The regions ranked as the most altered were those associated with Amide I and Amide II peaks (wavenumbers from 1746 to 1738 cm^−1^), followed by peaks falling in the “mixed region” [18,19] and associated with Amide III structure. The best IR regions individuated with this analysis were then used to produce a more refined hierarchical analysis, obtaining the same grouping of the samples as those obtained using the whole spectrum (Figure 2D), reaching a maximum heterogeneity value of about 7 a.u., lower than the one obtained with the full spectrum because fewer variables were used. Other compounds (e.g., fatty acids or carbohydrates) are also modified, although to a lower extent. In fact, the same analysis, performed on other IR regions, did not show the same differentiation as that obtained using the W_2_ and W_3_ regions.

### 2.2. ZA Alters the Expression of C. albicans Biofilm Proteins

T-test performed on Bradford assay data revealed that the global synthesis of protein in biofilm treated with ZA was higher in comparison to the control biofilm. Indeed, the treated biofilm showed 4.60 ± 0.47 μg proteins/μg biomass while, in the control biofilm, protein concentration was 3.56 ± 0.16 μg/μg biomass.

**Figure 3:**
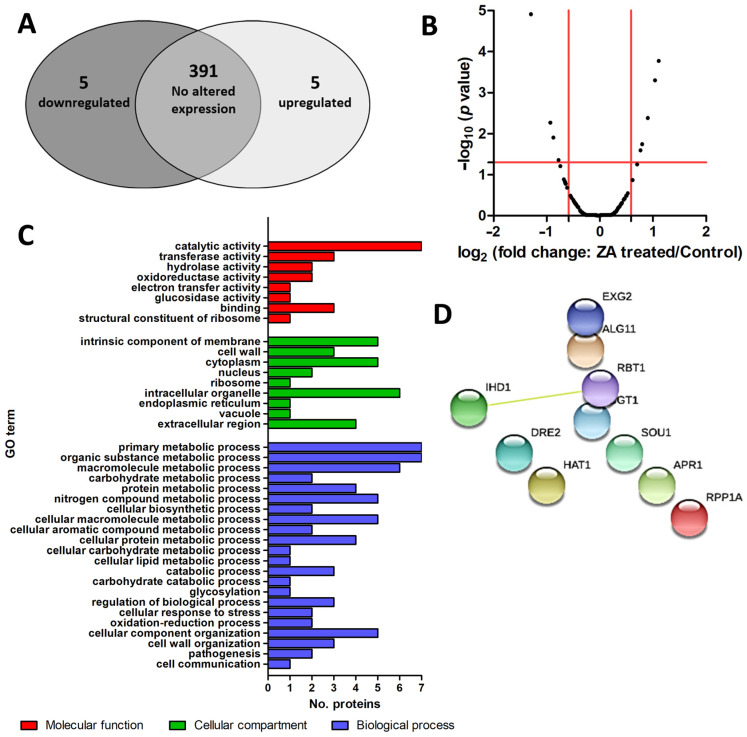
Venn diagram, Volcano plot, Panther Gene Ontology (GO)-slim functional analysis and String analysis of proteins differentially expressed in *C. albicans* biofilms upon 10 mg/L ZA treatment. (**Panel A**): Venn diagram with proteins significantly (a ± 1.5-fold change) and not significantly different in abundance between the control and treated samples. (**Panel B**): Volcano plot displaying the −log_10_ (*p* value) plotted against the log_2_ (fold change: treated/control). Points above the non-axial horizontal line represent proteins with significantly different abundances (*p* < 0.05). Points to the right of the right-most non-axial vertical line display protein with fold changes of treated/control ratio higher than 1.5, while points to the left of the left-most non-axial vertical line display protein with fold changes of treated/control ratio less than −1.5. (**Panel C**): Panther GO functional classification of proteins significantly upregulated and downregulated in the biofilm upon ZA treatment (molecular function: red; cellular compartment: green; biological process: blue). Second-level GO categories are reported. (**Panel D**): String analysis showing the predicted protein–protein interaction networks. Query proteins and first shell of interactor are reported.

nLC-HRMS identified 401 proteins in both the control and treated samples (Table S1). Among the proteins found, 10 proteins, which accounted for 2.7% of the total proteins, were found significantly different in abundance between the treated and the control samples (*t*-test analysis, *p* < 0.05; Table 1; Figure 3A). Indeed, in the biofilm upon ZA treatment, five proteins were found downregulated with changes between 1.7- and 2.5-fold and five proteins were found upregulated with changes between 1.2- and 2.2-fold. The Volcano plot in Figure 3B graphically displays the quantitative data.

Gene Ontology (GO) functional analysis was performed in order to obtain a global view of functions associated with downregulated and upregulated proteins. Proteins differentially expressed in the treated biofilm in comparison to the controls were clustered according to biological processes, molecular function and cellular compartment (Table S2, Figure 3C). GO functional analysis mapped all query proteins. Proteins differently expressed were components of membrane (five hits), cell wall (three hits), organelle (six hits) as well as cytoplasm (five hits) and were also present in the extracellular region (four hits). In terms of molecular function, upregulated and downregulated proteins were classified as responsible for catalytic activities (seven hits), including transferase (three hits), hydrolase (two hits) and glucosidase activity (one hit) activities, were involved in oxidoreductase activity (two hits), electron transfer activity (one hit) or were a structural constituent of ribosome (one hit) (Table S2, Figure 3C). Additionally, the most frequent of the biological processes were cellular (eight hits) and metabolic processes (eight hits) with proteins mostly involved in the metabolism of organic substances (seven hits) and macromolecules (six hits) as well as nitrogen compounds (five hits) and proteins (four hits) (Table S2, Figure 3C).

String analysis mapped all differently expressed proteins (Figure 3D). Analysis showed that these proteins were not connected, with the exception of cell wall protein Rbt1 (Rbt1) and induced during hyphae development protein (Ihd1) that displayed a functional link with a combined score of 0.469 (medium confidence score) [20].

## 3. Discussion

In this research, the mode of action of a seagrass secondary metabolite, i.e., ZA, against the fungal model *C. albicans* biofilm was elucidated by combining a metabolomic and proteomic approach.

FTIR analysis allowed us to discriminate among the physiological states of *C. albicans* cells throughout the biofilm and to characterize their metabolic changes in response to ZA. Furthermore, it confirmed the potential of this kind of approach as a primary screening to direct more detailed analyses toward dissecting the metabolomic compartment affected by the action of exogenous agents [21]. It also allows for comparative analyses of the same agent in different cellular models [22] and in different settings, using emerging ecological and environmental analysis protocols [23]. FTIR revealed that ZA altered the spectral regions associated with Amide I, Amide II and Amide III structures, indicating that proteins are one of the main targets of ZA action. Since FTIR analysis is primarily affected by the components of the cell envelope and, to a lesser extent, by the cytoplasm [24], these findings likely indicate that proteins associated with the membrane and the cell wall are the primary target of ZA. Therefore, the IR approach allowed us to direct the analysis to the entire proteome of *C. albicans* biofilm grown with and without ZA. FTIR analysis also indicated residual action against other compounds, but to a lesser extent, suggesting a need to concentrate the analyses on the proteins, while leaving to future work the study of the qualitative and quantitative effects on other compounds [25,26].

nLC-HRMS identified 10 proteins differentially expressed in the biofilm treated with ZA. Among these proteins, five were found downregulated and five upregulated in the treated biofilm in comparison to the control one.

Among the downregulated proteins, Rbt1 and Ihd1 had previously been reported to be necessary for biofilm formation and virulence. Indeed, both *rbt1* and *ihd1* take part in a network of eight genes, which have been linked to the core filamentation response, including germ tube formation followed by hyphal elongation [27,28,29]. Consistently, String analysis showed that Rbt1 and Ihd1 display a functional link with a medium confidence score.

Rbt1 was predicted to be a Glycosyl Phosphatidyl Inositol (GPI)-anchored protein, linked to the cell wall via a preformed GPI anchor sequence signal at its C-terminus that is added to the protein in the endoplasmic reticulum [30,31]. Experimental demonstration by Monniot et al. [32] showed the absence of Rbt1 at the surface of yeast cells and clarified that Rbt1 is dispersed along the hyphae after being deposited at the tip of the germ tube. Moreover, they showed the anchorage of Rbt1, both in the plasma membrane and the cell wall, and proved that Rbt1 is cell surface exposed and easily accessible on the exterior of the hyphae.

Plaine et al. [33] demonstrated that Rbt1 is necessary for the structural integrity and composition of the cell wall. Indeed, *rbt1−/−* mutants displayed increased vulnerability toward a number of cell-wall-perturbing agents and showed different phenotypes compared to the reference strain. Braun et al. [34] indicated that mutants lacking Rbt1 had significantly reduced virulence. They also suggested that Rbt1 might be involved in *C. albicans* adhesion and stable attachment of hyphae to the surface [34]. Similarly, Ene et al. [35] showed that *rbt1−/−* mutants were defective in biofilm formation. More recently, Monniot et al. [32] confirmed the involvement of Rbt1 in biofilm formation, showing that *C. albicans* Rbt1-overexpressing strains grew a biofilm with greater biomass than the control strain. *C. albicans* overexpressing Rbt1 also showed an increased cell surface hydrophobicity, therefore, correlating surface hydrophobicity to adhesiveness.

Monniot et al. [32] identified two key domains of Rbt1 implicated in the adhesion process to abiotic and biotic surfaces. The Rbt1 N-terminus acts as the substrate-binding domain and induces the clustering of different molecules of Rbt1, increasing adhesiveness and cell-to-cell interaction. Rbt1 deleted its N-terminus and was unable to promote the adhesion of recombinant *Saccharomyces cerevisiae* to polystyrene because the absence of the N-terminus made the domain involved in adherence no longer functional. At the C-terminus, Rbt1 contains two 42 amino acid-long motifs that mediate interactions between cells during biofilm development, without being implicated in the initial adherence. Additional data suggested that a peptide with high β-aggregation potential triggers the formation of hyphae aggregates and is involved in cell associations when *C. albicans* is in the hyphal form [32]. Indeed, when the full-length Rbt1 protein was expressed, a massive aggregation of hyphae was observed, in comparison to the *C. albicans* strain overexpressing the truncated protein, while the formation of aggregates was abolished in the *rbt1−/−* strain.

Ihd1 is a GPI-anchored cell wall and a trans-membrane protein with a hydrophobic domain at both its C- and N-terminal ends [36].

Hameed et al. [37] showed that *ihd1−/−* mutants were more sensitive towards drugs and salts perturbing cell wall formation, confirming that Ihd1 is involved in cell wall biogenesis and integrity.

Together with Rbt1, Ihd1 takes part in the core filamentous response [27,28,29]. Hameed et al. [37] showed that, in comparison to the control strain, mutants exhibited defective hyphal growth, smaller colony size, a shortened cell cycle, a decreased budding index and budding size and an altered bud shape that was oval, elongated and speared. However, Martin et al. [27] observed that the deletion of ihd1 did not affect the ability to form hyphae, indicating that the protein is not required for the yeast to hyphae transition, but it is likely involved in the late filamentation process.

McCall et al. [38] demonstrated that *C. albicans* Δ*ihd1* cells had significantly reduced initial attachment and adhesion maintenance. Indeed, deletion of *ihd1* in *C. albicans* resulted in a near complete loss of biofilm development, with a ten-fold decrease in adhesion and a two-fold increase in dispersion during surface attachment. Accordingly, Cabral et al. [39] showed that overexpression of Ihd1 significantly increased adherence of *C. albicans* cells, confirming the role of this protein in cell adhesion.

Villa and colleagues [5] observed that when *C. albicans* biofilm was treated with ZA, biofilm was reduced by at least 70% and showed morphostructural alterations with microcolonies of predominantly yeast forms and very few filamentous structures of abnormal length, greatly reducing biofilm thickness, and this also occurred when ZA was added after the adhesion phase. This is fully consistent with a putative interaction of ZA with both Rbt1 and Ihd1 that, being reduced in expression by ZA, decreases biofilm formation and limits hyphal development. Since Rbt1 and Ihd1 demonstrated a pivotal role in the adhesion processes and hyphal formation, they represent an attractive opportunity for designing new biofilm inhibitors based on these targets. Indeed, adhesion processes and the ability to switch between yeast and hyphal growth forms are considered of major importance in the first steps of *C. albicans* colonization and biofilm formation and are the most discussed virulence attributes.

Consistent with the fact that ZA might affect cell wall structure, the glucan 1,3-beta-glucosidase (Exg2) and alpha-1,2-mannosyltransferase (Alg11) were found downregulated in the treated biofilm.

β-glucosidases hydrolyze glycosidic bonds in oligosaccharides. In the fungal outer cell wall, these enzymes locally modify cell wall structure and contribute to the turnover of the most abundant cell wall polysaccharide β-glucans by cleaving glycosidic bonds [40]. By perturbing β-glucan biosynthetic processes, ZA might remodel the cell wall, leading to changing the host *C. albicans* interaction.

β-glucans are the point of attachment for many cell wall mannoproteins. These are glycosylated proteins that dominate the cell wall’s outer skeletal layer [41]. Protein mannosylation occurs in both the endoplasmic reticulum and Golgi apparatus during protein synthesis. Alg11, here found with a reduced expression, is a mannosyltransferase that catalyzes the last mannosylation step at the cytoplasmic side of the endoplasmic reticulum, leading to Man4GlcNAc2-PP-dolichol and Man5GlcNAc2-PP-dolichol oligosaccharides. These oligosaccharides are required for the assembly, among others, of the cell walls’ N-linked glycosylated proteins [42,43]. It has been reported that loss of the *alg11* in *S. cerevisiae* weakened cell walls due to defective glycosylation. In addition, the deletion of *alg11* was not lethal but led to a severely slow-growing phenotype [43]. In this work, Alg11 was found downregulated in the treated biofilm, confirming a key role of ZA in interfering with the glycosylation process and in perturbing the localization of mannans in the wall, with effects on the entire cell wall and fungal virulence.

Type B histone acetyltransferase (HATs-B) is one the key players involved in the deposition of newly synthesized histones into chromatin. Histone acetyltransferase type B catalytic subunit 1 (Hat1) was the first type of HATs-B identified and was found conserved throughout the eukaryotic kingdom. In the present research, we found that Hat1 was upregulated in the biofilm treated with ZA.

Together with the Hat2 subunit, Hat1 acetylates free histone H4 within the cytoplasm and the histone acetylated in the cytoplasm is transported into the nucleus and it is incorporated into the nucleosomes [44,45,46]. After the addition of a further subunit in the nucleus, the NuB4 complex is formed. Indeed, Hat1 is the catalytic subunit of the well-known HAT-B NuB4 complex of *C. albicans* [47].

It has been reported that, when *C. albicans* recognizes oxidative stress signals, the expression levels of the oxidative-stress-responsive genes are induced by NuB4 [46]. Tscherner et al. [45] showed that the loss of Hat1 markedly increased resistance to some oxidizing agents, by accelerating the transcription of genes encoding for proteins involved in the response to oxidative stress. Therefore, a specific role for Hat1 in the regulation of oxidative stress resistance was proved. Furthermore, it was shown that *C. albicans hat1−/−* mutant cells were defective in maintaining the yeast morphology and easily switched from yeast-like to hyphal morphology, as a consequence of spontaneous DNA damage accumulation [45]. A strong upregulation of the typical hyphae-induced genes in mutants was observed, demonstrating a fully active hyphal transcriptional program [45,48].

Indeed, Hat1 expression might be induced when *C. albicans* recognizes oxidative stress signals and responds by changing the chromatin structure. As Hat1 is also a negative regulator of hyphae formation, its upregulation by ZA may strongly reduce the transcription of typical hyphae-inducing genes, favoring the yeast morphology at the expense of hyphal phenotype, as observed by Villa et al. [5].

In line with these observations, ZA affects biofilm formation of the bacterium *Escherichia coli* by fine-tuning the threshold level for oxidative stress, by targeting the enzyme quinone oxidoreductase WrbA. In *E. coli*, the loss of WrbA leads to an ROS-sensitive phenotype and reductions in biofilm thickness, biofilm-dwelling cells, matrix polysaccharide content and H_2_O_2_ tolerance was observed [14,15].

Villa et al. [5] proved that 10 mg/L ZA increased the activity of the antimicrobial agents chlorhexidine, chlorine, hydrogen peroxide and cis-2-decenoic acid. According to our previous results, a critical role of Hat1 in fungal virulence and pathogenesis has been reported. It was found that *hat1−/−* mutants were more resistant to killing by innate immune cells, displayed more persistence in a murine infection model and were more resistant toward different azole drugs, because of a defect in chromatin assembly [45,48]. As a consequence, upregulation of Hat1 in ZA-treated *C. albicans* biofilm likely leads to enhanced sensitivity toward antifungal drugs, especially those having an ROS-mediated mode of action and being defective in virulence.

The Fe-S cluster assembly protein DRE2 (Dre 2) is involved in the biogenesis of cytosolic Fe-S cluster protein implicated in the extramitochondrial Fe-S cluster assembly machinery [49]. These proteins take part in several pathways essential for the viability of each living cell, including metabolic pathways, DNA maintenance and protein translation [50]. The innate chemistry of their Fe–S cofactors makes these regulatory proteins ideal for sensing environmental signals, including, among others, ROS and redox cycling compounds, to subsequently mediate an adaptive response [51]. Because of their capacity to delocalize electrons over both S and Fe ions, Fe–S clusters can be easily oxidated by molecules, such as oxygen (O_2_),redox-cycling drugs and ROS, such as hydrogen peroxide (H_2_O_2_) and superoxide (O_2_^−^), potentially leading to cluster conversion or complete cluster loss [51,52]. Interestingly, this protein was found to be reduced when the biofilm was treated with ZA, confirming a possible role of ROS in ZA’s mode of action.

Other proteins were also found overexpressed in *C. albicans* biofilm upon ZA treatment and were associated with the stress response.

The structural gene *sou1* encodes for a short-chain dehydrogenase. This enzyme catalyzes the first step in the L-sorbose metabolic pathway by converting this sugar to fructose-6-phosphate on the glycolytic pathway [53]. As L-sorbose is not naturally present in the niches occupied by *C. albicans*, these genes may be involved in a general stress response mechanism [54]. Accordingly, Sou1 was found overexpressed in *C. albicans* cells under osmotic stress [55]. However, the precise function of Sou1 is not known [53].

Apr1 is involved in vacuolar proteolysis. This is very important when yeast is under stress conditions [56]. Amri Saroukolaei et al. [56] demonstrated that *C. albicans* displays increased amounts of Apr1 and enzyme activity upon entering a new environment, as a form of adaptation to the new conditions to survive.

60S acidic ribosomal protein P1-alpha (Rpp1a) and mRNA-capping enzyme subunit alpha (Cgt1) involved in the synthesis of proteins were also found upregulated in biofilm upon ZA treatment, as a result of transcript increased levels.

Rpp1a is a component in the ribosome and it belongs to a large ribonucleoprotein complex responsible for the synthesis of proteins in the cell. It plays an important role in the elongation step of protein synthesis. Cgt1 is involved in the acquisition of the 5′ cap, the earliest modification event during eukaryotic mRNA synthesis, and it is essential for fungal viability [57,58]. Interestingly, it has been reported that *S. cerevisiae* bud morphology defects can partially be attributed to impaired ribosome function [59,60]. Accordingly, Cgt1-deficient *C. albicans* mutants exhibited much larger and irregularly shaped colonies with a tentacle-like structure, in contrast to the parental wild-type strain that produced circular and smooth colonies [57].

## 4. Materials and Methods

### 4.1. Candida albicans Strain and Growth Conditions

The well-characterized *C. albicans* ATCC MYA-2876 was used as a model system for fungal biofilms. The strain was stored at −80 °C in suspensions containing 20% glycerol and 2% peptone and was routinely grown at 30 °C for 24 h in the yeast nitrogen base broth supplemented with 50 mM of glucose (YNBg, Sigma Aldrich, St. Louis, MI, USA). After being routinely grown, *C. albicans* cells were washed three times in Phosphate-Buffer Saline (PBS, Sigma Aldrich) and were used to grow biofilms. A light microscope (Leica DM4000 M, Leica Microsystems, Wetzlar, Germany) and a Thoma counting chamber were used to quantify *C. albicans* cell concentration.

### 4.2. Biofilm Growth in the CDC Reactor

*C. albicans* biofilms were grown without and with 10 mg/L ZA at 30 °C on polycarbonate coupons in Center for Disease Control Bioreactors (CDC reactor, Biosurface Technologie, Bozeman, MO, USA) as described by Villa et al. [5]. Briefly, the bioreactor was inoculated with 400 mL of sterile YNBg medium with the addition of 1 mL of pre-washed overnight culture containing 10^7^ cells. The culture was grown at 30 °C for 24 h. After 24 h of the adhesion phase, the peristaltic pump was started and sterile 20% YNBg was continuously pumped into the reactor at a rate of 250 mL/h. A magnetically driven stir bar was used to continuously mix the bulk fluid. After 48 h of the dynamic phase, coupons were removed from the bioreactor, gently washed with PBS and processed to be analyzed.

### 4.3. Fourier-Transform Infrared Spectroscopy (FTIR) Study

#### 4.3.1. Sample Preparation

Collected coupons were transferred to 5 mL of PBS. Biofilm was removed from the coupon surface by 1 min vortex mixing, 2 min sonication (50% amplitude, in water bath; Branson 3510, Branson Ultrasonic Corporation, Dunburry, CT, USA) followed by another 1 min of vortex mixing. To reduce bacterial aggregates, cell suspensions were homogenized by two 30 s cycles at 14,500 rpm (T 10 basic Ultra-Turrax, IKA, Rawang, Malaysia), vortexed for 30 s and passed through a syringe needle of a 0.4 mm section.

#### 4.3.2. FTIR Analysis

FTIR experiments were carried out according to Corte et al. [21]. Briefly, 35 μL of each sample was loaded and dried on a silicon multiwall plate and FTIR measurements were performed in transmission mode, using a TENSOR 27 FTIR spectrometer, equipped with an HTS-XT accessory for rapid automation of the analysis (Bruker Optics GmbH, Ettlingen, Germany). Background was run with both the wall empty and loaded with PBS. Spectra were recorded in a range between 4000 and 400 cm^−1^, with a 4 cm^−1^ spectral resolution and setting 256 scans per sample. Quality test, baseline correction, background subtraction, vector normalization and the calculation of the first and second derivatives of spectral values were carried out by using the software OPUS version 6.5 (Bruker Optics GmbH, Ettlingen, Germany).

Four technical replicates were prepared for each sample and four instrumental replicates were run. The experiment was repeated three times.

#### 4.3.3. Data Analysis

Each single spectrum was normalized in order to have a range spanning from 0 to 1. Average spectra of each fraction of treated and control biofilm were calculated.

Hierarchical cluster analysis was performed considering the whole spectrum as well as four separate spectral regions: fatty acids (W_1_) from 3000 to 2800 cm^−1^, amides (W_2_) from 1800 to 1500 cm^−1^, mixed region (W_3_) from 1500 to 1200 cm^−1^ and carbohydrates (W_4_) from 1200 to 900 cm^−1^.

Correlation analysis of IR data was performed with MetaboAnalyst 5.0 [61]. Data were filtered based on Interquartile Range and scaled by Pareto scaling. The analysis was carried out to highlight which wavenumbers were altered to a greater extent by zosteric acid exposition, setting the Euclidean correlation method and Ward clustering algorithm. Wavenumbers were, therefore, selected according to the VIP scores from Partial Least Square-Discriminant Analysis (PLS-DA). Since the average of the squared VIP score is equal to 1, the “greater than one” rule was used as a criterion for variable selection.

### 4.4. High-Resolution Mass Spectrometry (nLC-HRMS) Study

#### 4.4.1. Protein Extraction

Collected coupons were gently washed with PBS and biofilms were scratched and suspended in 6 M urea to a final concentration of 125 μg/mL. Biofilms were disaggregated by vortex mixing (15 min) and sonication (two 30 s sonication cycles at 22 μm amplitude in Soniprep 150). Samples were transferred in screw-cap 2 mL vials containing approximately 100 μL of glass beads (diameter <106 μm and between 425 and 600 μm, Sigma Aldrich). A Precellys bead beater (Bertin Instrument, Montigny-le-Bretonneux, France; 6 cycles of 30 s at 6500 rpm, with a 30 s period of cooling between cycles) was used for mechanical disruption of cells. Samples were centrifuged at 11,000× *g* for 30 min at 4 °C and the supernatants containing proteins released from the broken cells were recovered. The Bradford assay [62] was used to quantify protein concentration (bovine serum albumin as standard). T-test analysis (GraphPad Prism Software, version 8.0.0, San Diego, CA, USA) was applied to statistically evaluate any significant differences among the biofilm protein concentrations. Statistically significant results were decided by *p*-values < 0.05.

#### 4.4.2. Sample Preparation

A suitable amount of proteins in 50 mM NH₄HCO₃ were reduced with dithiothreitol (5 mM final concentration, Sigma-Aldrich, St. Louis, MO, USA) for 30 min at 55 °C. Iodoacetamide (Sigma-Aldrich) was added to a final concentration of 15 mM and samples were incubated for 30 min in the dark at room temperature. Proteins were digested with trypsin (Promega Italia SRL, Milano, Italy) with an enzyme–protein ratio of 1:20 at 37 °C overnight. The addition of trifluoroacetic acid (Sigma Aldrich, USA, final concentration of 0.5%) was used to stop the reaction. The digested samples were further purified and concentrated by 0.2 μL C-18 resin ZipTip (Millipore, Burlington, MA, USA) in order to increase the quality of instrumental analysis.

#### 4.4.3. nLC-HRMS Analysis

One µg total of the sample was analyzed by a Dionex Ultimate 3000 nano-LC system (Sunnyvale, CA, USA) connected to an Orbitrap Fusion™ Tribrid™ Mass Spectrometer (Thermo Scientific, Bremen, Germany) equipped with a nano electrospray ion source. Peptide mixtures were pre-concentrated onto an Acclaim PepMap 100–100 μm × 2 cm C18 (Thermo Scientific) and separated on EASY-Spray column, 15 cm × 75 μm ID packed with Thermo Scientific Acclaim PepMap RSLC C18, 3 μm, 100 Å. The flow rate was 300 nL/min and the temperature was set to 35 °C. Mobile phases were 0.1% formic acid (FA) in water (buffer A) and 0.1% FA in water/acetonitrile with 2/8 ratio (buffer B). The elution gradient was from 96% buffer A to 95% buffer B for 110 min. MS spectra were collected over an m/z range of 375–1500 Da at 120,000 resolutions, operating in data-dependent mode, cycle time of 3 s between master scans. Higher-energy collision dissociation was performed by collision energy set at 35 eV and positive polarity. The experiment was repeated three times and each sample was run in three technical replicates.

#### 4.4.4. Bioinformatic, Statistical and Functional Annotation Analysis

A protein database using SEQUEST algorithm in Proteome Discoverer software version 2.2 (Thermo Scientific) for peptide/protein identification was used to analyze the Thermo raw data. The searches were performed against Uniprot KnowledgeBase (KB)/Swiss-Prot (taxonomy ID 237561—*Candida albicans*). The minimum peptide length was set to 6 amino acids and enzymatic digestion with trypsin was selected, with maximum of 2 missed cleavages. A precursor mass tolerance of 8 ppm and fragment mass tolerance of 0.02 Da were used; Met loss + acetylation (N-term) and oxidation (M) were used as dynamic modifications and carbamidomethylation (C) as static modification.

In order to determine the peptide false-discovery rate, a decoy database search was performed with the percolator module. The false-discovery rates at the protein and peptide level were set to 0.01 for highly confident peptide-spectrum matches and 0.05 for peptide-spectrum matches with moderate confidence.

Protein quantification was based on label-free quantification (LFQ). The fold changes in the level of the proteins were evaluated by comparing the mean LFQ intensities among the treated and control samples. A protein was considered differentially expressed if the difference was statistically significant (*p* < 0.05), the fold change minimum was ±1.5 and it was identified with a minimum of two peptides.

Volcano plot scaling was performed by transforming the fold changes into log2 and the *p*-value into -log10 using the GraphPad Prism version 8.0.0 for Windows, GraphPad Software, San Diego, CA, USABiological processes, molecular function and cellular compartments of differentially abundant proteins were studied by GO analysis via the PANTHER Classification System [63]. Protein network analysis was performed using String [64].

## 5. Conclusions

The results of this study partially cover the existing gap in the knowledge of ZA’s mechanism of action against fungi, enlarging the present perspective for its widespread use as a powerful antibiofilm agent, especially in those challenging fields where fungal biofilms are very recalcitrant and when the use of traditional antimicrobial agents is limited for safety reasons. Additionally, this study provided insight into the major *C. albicans* pathways associated with the biofilm phenotype.

FTIR analysis confirmed that proteins are the primary targets of ZA and directed research toward the investigation of the entire proteome of *C. albicans* treated or not treated with ZA.

Proteins involved in the biogenesis, structure and integrity of cell walls as well as adhesion and stable attachment of hyphae were found downregulated, and these findings make sense in terms of the morphostructural alterations previously observed by the same authors in the *C. albicans* biofilm under ZA treatment [5]. Some proteins involved in stress response, including oxidative stress, were also found overexpressed as a consequence of fungal adaptation to hostile environments.

The variation in the protein patterns indicated that the presence of ZA acts as a stress factor on the sessile cells, which seems to discourage the colonization of the surface. It is likely that ZA is sensed as an environmental cue, leading to the rapid alteration of the yeast-to-hypha transcriptional program by a mechanism involving chromatin modifiers and triggered by ROS production.

The challenge now is to use this knowledge to design novel multi-target antibiofilm compounds with enhanced activity. In this direction, modern drug discovery based on computational approaches has the power to identify potential modulators of multiple targets from millions of compounds without chemical synthesis and biological testing. Indeed, by modulating multiple selected targets simultaneously additive or synergistic effects could be obtained [65]. This implies that the antibiofilm effect is attained at lower doses and, consequently, compounds with improved performances are expected to be generated. Undoubtedly, the critical task is to establish the right degree of modulation for each target in order to avoid unwanted interactions [65]. In addition, the quantitative structure–activity relationship (QSAR) can correlate ZA molecular structure with its biological antibiofilm activities, selecting the molecular descriptors that predict drug–target interactions.

## Figures and Tables

**Table 1:** Differentially expressed proteins in biofilms upon ZA treatment. Fold changes with respect to the control were calculated by dividing the label-free quantification (LFQ) mean intensity value of the treated samples by that of control samples.

Accession	Gene Name	Description	ZA Treated/ Control
**Downregulated**			
Q59TP1	*rbt1*	Cell wall protein Rbt1	0.41
Q5A8I8	*ihd1*	Induced during hyphae development protein	0.52
C4YL88	*dre2*	Fe-S cluster assembly protein	0.52
Q5AIA1	*exg2*	Glucan 1,3-beta-glucosidase 2	0.55
Q59S72	*alg11*	GDP-Man:Man(3)GlcNAc(2)-PP-Dol alpha-1,2-mannosyltransferase	0.58
**Upregulated**			
Q9HFQ7	*rpp1A*	60S acidic ribosomal protein P1-A	1.70
P10977	*apr1*	Vacuolar aspartic protease	1.74
Q59VF4	*hat1*	Histone acetyltransferase type B catalytic subunit	1.87
P87219	*sou1*	Sorbose reductase	2.05
P78587	*cgt1*	mRNA-capping enzyme subunit alpha	2.16

## Data Availability

The data presented in this study are available in the Supplementary Material.

## Supplementary Materials

The following supporting information can be downloaded at: https://www.mdpi.com/article/10.3390/ijms232214067/s1.
